# Immunogenicity and safety of fractional doses of 17D-213 yellow fever vaccine in HIV-infected people in Kenya (YEFE): a randomised, double-blind, non-inferiority substudy of a phase 4 trial

**DOI:** 10.1016/S1473-3099(23)00114-7

**Published:** 2023-08

**Authors:** Derick Kimathi, Aitana Juan-Giner, Benedict Orindi, Kyra H Grantz, Ndeye S Bob, Stanley Cheruiyot, Mainga Hamaluba, Naomi Kamau, Gamou Fall, Moussa Dia, Moses Mosobo, Felix Moki, Kenneth Kiogora, Oscar Chirro, Alexander Thiong'o, Jane Mwendwa, Andrew Guantai, Henry K Karanja, John Gitonga, Daisy Mugo, Kelly Ramko, Ousmane Faye, Eduard J Sanders, Rebecca F Grais, Philip Bejon, George M Warimwe

**Affiliations:** a Kenya Medical Research Institute—Wellcome Trust Research Programme, Kilifi, Kenya; b Centre for Tropical Medicine & Global Health, University of Oxford, Oxford, UK; c Epicentre, Paris, France; d Department of Biology and Emerging Pathogens Institute, University of Florida, Gainesville, FL, USA; e Department of Epidemiology, Johns Hopkins Bloomberg School of Public Health, Baltimore, MD, USA; f Institut Pasteur Dakar, Dakar, Senegal

## Abstract

**Background:**

Evidence indicates that fractional doses of yellow fever vaccine are safe and sufficiently immunogenic for use during yellow fever outbreaks. However, there are no data on the generalisability of this observation to populations living with HIV. Therefore, we aimed to evaluate the immunogenicity of fractional and standard doses of yellow fever vaccine in HIV-positive adults.

**Methods:**

We conducted a randomised, double-blind, non-inferiority substudy in Kilifi, coastal Kenya to compare the immunogenicity and safety of a fractional dose (one-fifth of the standard dose) versus the standard dose of 17D-213 yellow fever vaccine among HIV-positive volunteers. HIV-positive participants aged 18–59 years, with baseline CD4^+^ T-cell count of at least 200 cells per mL, and who were not pregnant, had no previous history of yellow fever vaccination or infection, and had no contraindication for yellow fever vaccination were recruited from the community. Participants were randomly assigned 1:1 in blocks (variable block sizes) to either a fractional dose or a standard dose of the 17D-213 yellow fever vaccine. Vaccines were administered subcutaneously by an unblinded nurse and pharmacist; all other study personnel were blinded to the vaccine allocation. The primary outcome of the study was the proportion of participants who seroconverted by the plaque reduction neutralisation test (PRNT_50_) 28 days after vaccination for the fractional dose versus the standard dose in the per-protocol population. Secondary outcomes were assessment of adverse events and immunogenicity during the 1-year follow-up period. Participants were considered to have seroconverted if the post-vaccination antibody titre was at least 4 times greater than the pre-vaccination titre. We set a non-inferiority margin of not less than a 17% decrease in seroconversion in the fractional dose compared with the standard dose. This study is registered with ClinicalTrials.gov, NCT02991495.

**Findings:**

Between Jan 29, 2019, and May 17, 2019, 303 participants were screened, and 250 participants were included and vaccinated; 126 participants were assigned to the fractional dose and 124 to the standard dose. 28 days after vaccination, 112 (96%, 95% CI 90–99) of 117 participants in the fractional dose group and 115 (98%, 94–100) of 117 in the standard dose group seroconverted by PRNT_50_. The difference in seroconversion between the fractional dose and the standard dose was –3% (95% CI –7 to 2). Fractional dosing therefore met the non-inferiority criterion, and non-inferiority was maintained for 1 year. The most common adverse events were headache (n=31 [12%]), fatigue (n=23 [9%]), myalgia (n=23 [9%]), and cough (n=14 [6%]). Reported adverse events were either mild (182 [97%] of 187 adverse events) or moderate (5 [3%]) and were self-limiting.

**Interpretation:**

Fractional doses of the 17D-213 yellow fever vaccine were sufficiently immunogenic and safe demonstrating non-inferiority to the standard vaccine dose in HIV-infected individuals with CD4^+^ T cell counts of at least 200 cells per mL. These results provide confidence that fractional dose recommendations are applicable to populations with high HIV prevalence.

**Funding:**

Wellcome Trust, Médecins Sans Frontières Foundation, and the UK Department for International Development.

## Introduction

Yellow fever, a haemorrhagic arboviral disease, is a re-emerging disease of public health importance. It has been estimated that 10–15% of infections are severe, requiring intensive care support, and associated with a high fatality rate.^1^ Despite the existence of a safe and effective vaccine, yellow fever outbreaks have increasingly been reported in sub-Saharan Africa and tropical South America.^2^ This is partly due to low vaccine coverage owing to a global shortage in vaccine supply. In a preceding study,^3^ we assessed all four WHO-prequalified vaccines in an adult population and showed that fractional doses are safe and sufficiently immunogenic and can be used during outbreaks, but this evidence might not be generalisable to populations living with HIV. There is a substantial burden of HIV and associated acquired immunodeficiencies in sub-Saharan Africa where yellow fever is endemic. For example, in 2021, about 26 million HIV-infected individuals (more than two-thirds of the global burden) lived in sub-Saharan Africa.^4^

**Research in context**

**Evidence before this study**

We searched ClinicalTrials.gov and The international Clinical Trials Registry Platform for trials registered between database inception and Feb 4, 2023. Of 103 trials on yellow fever, seven were in HIV-positive participants, none of which assessed fractional dosing of yellow fever vaccination prospectively. A systematic review and meta-analysis examined the immunogenicity and safety of fractional doses and noted that there were no data from randomised clinical trials assessing use of fractional doses among HIV-infected individuals.

**Added value of this study**

To our knowledge, we assessed, for the first-time, fractional doses of the yellow fever vaccine in HIV-infected adults without signs of clinical disease in coastal Kenya. The study evaluated the immunogenicity and safety of the fractional doses of the yellow fever vaccine at 10 days, 28 days, and 1 year after vaccination. The results show that fractional doses met non-inferiority 28 days after vaccination, and that protection was maintained at 1-year follow-up. 10 days after vaccination, however, seroconversion and neutralising antibody levels were significantly lower in the fractional dose arm. There were no safety concerns in this population.

**Implications of all the available evidence**

The results from this study increase confidence that one-fifth fractional doses of yellow fever vaccines can be used in HIV-infected individuals. The data further support evidence-based guidelines for HIV-infected individuals. Studies evaluating the longevity of vaccine-induced immunity are needed to inform policies for wider use of the dose-sparing strategy.

In Kenya, an estimated 1·3 million people were living with HIV in 2018, with about 37 000 new infections per year.^5^ In the coastal region of Kenya, where we conducted our previous study of the four WHO-prequalified vaccines,^3^ HIV prevalence was estimated at 4·9% during 2018.^5^ Flaviviruses, such as dengue virus cause sporadic outbreaks in the region,^6^ but yellow fever virus transmission has not been reported on the coast of Kenya. However, yellow fever vaccination is included in the routine Expanded Program of Immunisation for populations in the Kenyan Rift Valley region (located 700 km northwest of the coastal region) where yellow fever transmission is endemic.

WHO-prequalified yellow fever vaccines are live attenuated virus vaccines derived from the 17D strain (substrains 17DD and 17D-204 and 17D-213, a substrain of 17D-204). Vaccines are formulated to contain a minimum potency of 1000 IU per dose, but average doses are much higher than the recommended minimum.^7^ Yellow fever vaccination is contraindicated in people with severe immunodeficiencies including people with severe HIV infection and AIDS in principle, because of its potential for virulence and ability to cause severe adverse events including yellow fever vaccine-associated viscerotropic diseases and neurotropic disease.^8^ WHO recommends yellow fever vaccination for HIV-infected people aged 9 months or older who are asymptomatic and with CD4^+^ T cell counts of at least 200 cells per mL.^8^ This recommendation applies to the routine Expanded Program of Immunisation, for travel, and reactive vaccination campaigns during epidemics and is supported by data on immunogenicity and safety of the yellow fever vaccine among HIV-infected people with CD4^+^ T-cell counts of at least 200 cells per mL.^9^

Although standard doses of the yellow fever vaccine are considered safe in people living with HIV, vaccination might result in delayed and lower rates of seroconversion and lower immunogenicity, efficacy, and herd immunity compared with HIV-negative populations.^10^ Higher CD4^+^ T-cell counts and lower HIV RNA levels at the time of immunisation correlate with a better immune response to the yellow fever vaccination.^11^ Infection with HIV has been thought to lower the duration of protection in first-time vaccinees. Evidence regarding seroconversion and duration of humoral and cellular immunity after vaccination and revaccination in HIV-infected individuals is limited to a few studies and no studies have assessed the use of fractional doses in this population,^12^ which limits rational development of comprehensive yellow fever vaccination guidelines specific for this population.^10^

Following the preceding study assessing all four WHO-prequalified yellow fever vaccines,^3^ we conducted a substudy to assess the immunogenicity and safety of fractional doses among HIV-positive individuals in coastal Kenya^13^ and generate further evidence for the use of fractional dosing in this population.

## Methods

### Study design and participants

This randomised, double-blind, non-inferiority study of 17D-213 yellow fever vaccination in HIV-infected individuals was a substudy of a trial conducted between Nov 6, 2017, and Feb 21, 2018, evaluating all four WHO-prequalified yellow fever vaccines in the general population.^3^ This larger study found that fractional doses from all the vaccines were safe and met the non-inferiority criteria for seroconversion compared with standard doses.^3^ Following these results, and considering supply and production capacity of the different manufacturers, the study Data and Safety Monitoring Board (DSMB), as prespecified in the protocol, recommended one of the four WHO prequalified vaccines for evaluation in this substudy. The Chumakov vaccine (17D-213), manufactured by the Institute of Poliomyelitis and Viral Encephalitis (Moscow, Russia), was selected. Of the vaccine batches available, we chose the batch with the closest potency to the manufacturer's minimum release specification (lot 598). This substudy received ethical approval from the Kenya Medical Research Institute Scientific and Ethics Review Unit, the Oxford Tropical Research Ethics Committee, and the WHO Ethics Review Committee, and regulatory approval from the Pharmacy and Poisons Board of Kenya. The clinical trial was conducted in accordance with ICH Good Clinical Practice guidelines.^14^

Participants were recruited from comprehensive care centres in Mtwapa and Malindi in Kilifi County on the coast of Kenya—17 km and 115 km northeast of Mombasa, respectively. A community engagement plan for the study was developed at each of the comprehensive care centre sites. In this plan, we involved the local subnational health management teams, local administration, comprehensive care providers at the centres, and the members attending the comprehensive care clinics. Potential participants were identified and sensitised for the trial. Willing volunteers provided written informed consent before any study-specific procedures were done to assess their eligibility.

Individuals aged 18–59 years, with no contraindication for yellow fever vaccination or previous history of yellow fever infection or vaccination, and with CD4^+^ T-cell counts of at least 200 cells per mL were included. We excluded pregnant volunteers (as determined by urine test), those intending to travel to countries requiring yellow fever vaccination, and those intending to move out of the study area before the end of the study.

### Randomisation and masking

Participants were randomly assigned 1:1 to receive either a standard dose or a fractional dose (one-fifth the standard dose) of the 17D-213 YF vaccine lot 598 with a release potency of around 67 608 IU per dose. The potency of the vaccine was confirmed at the National Institute for Biological Standards and Control (Potters Bar, UK; appendix p 2).

Randomisation was done in non-disclosed variable block sizes of 6 or 8. The randomisation sequence was pre-generated and concealed in a scratch-off booklet by an independent firm (DiagnoSearch LifeSciences, Mumbai, India). Participants and study personnel following safety and immunological outcomes were blinded to the allocations throughout the study. The vaccines were prepared at the start of each vaccination day by the unblinded personnel (the vaccinating nurse and pharmacist) on the basis of manufacturer's instructions and were kept at 2–8°C until administration. Vaccines were administered subcutaneously. Standard doses were dispensed using 0·5 ml auto-disable syringes (standard subcutaneous needle size 25G × 3/4”) at about a 45° angle. Fractional doses were dispensed using 0·1 mL auto-dosing syringes (needle size 26G × 3/8”) at about a 90° angle to achieve subcutaneous delivery.^15^ The syringes were the same in appearance and only the needle size varied. The volume in the syringe was masked using opaque tape. Upon vaccination, the participant was observed for 30 minutes to assess for any immediate reactions.

### Procedures

Upon recruitment, baseline blood samples were collected to assess CD4^+^ T-cell counts. These were measured as per the Government of Kenya guidelines.^16^ Although viral RNA might be a correlate of immunogenicity and is an established predictor of HIV/AIDS progression, we did not test for HIV viraemia in this study. The trial objectives related to the effect of CD4^+^ T-cell counts on immunogenicity, judging this to be a more direct measure of the effector immune response.

4 mL venous blood samples were collected before vaccination to assess presence of yellow fever antibodies in serum from previous infection or vaccination, 10 days after vaccination to assess rapidity of immunological protection, 28 days after vaccination to assess immunogenicity (primary outcome), and 1 year after vaccination to assess the longevity of the antibodies. Whole blood samples were separated by centrifugation to collect serum within 4 h of collection and stored at –80°C at the Kenya Medical Research Institute—Wellcome Trust Research Programme's repository and later shipped to Institute Pasteur de Dakar (Dakar, Senegal) for virus neutralisation tests.

Immunogenicity was tested using a standardised plaque reduction neutralisation test (PRNT), the gold standard for evaluating functional immunity in response to yellow fever vaccination.^2^ This study used the PRNT_50_ as a primary endpoint and PRNT_90_ as a secondary outcome.^13^ These assays were conducted at the WHO yellow fever regional reference laboratory at Institut Pasteur de Dakar, where there is an established PRNT assay using the 17D-204 vaccine strain that has previously been described.^3^, ^17^

Any adverse event regardless of seriousness was actively monitored and recorded within 30 minutes after vaccination, at the scheduled study visits (day 10, day 28), and any unscheduled visits within 28 days of vaccination. Participants were also advised to report any substantial health events to the research clinic during the entire duration of the trial. Serious adverse events^18^ and suspected unexpected serious adverse reactions were monitored throughout the study period. Reporting of the safety-related events was standardised by coding using Medical Dictionary for Regulatory Activities version 20.0. The events were assessed for the degree of certainty with which they could be attributed to vaccination on the basis of previous reports of similar events after yellow fever vaccination and the temporal association of the event with vaccination. Events were classified as related if there was a reasonable possibility that vaccination contributed to the adverse event.

### Outcomes

The primary outcome was the proportion of participants who seroconverted by 28 days after vaccination for the fractional dose compared with the standard dose in the per-protocol population. Seroconversion was defined as a four-fold or greater increase in neutralising antibody titre between pre-vaccination and post-vaccination samples as measured by PRNT_50_. Seronegative samples (ie, PRNT_50_ below the limit of quantification [LOQ], <1:10) were assigned a nominal value of LOQ/2 such that a four-fold increase for a participant who was less than 1:10 at baseline corresponded with a titre of 20. Secondary outcomes included geometric mean titres (GMTs) and geometric mean fold increase (GMFI) and their ratios relative to baseline and assessment of adverse events. Immunogenicity outcomes were also assessed at 10 days and 1 year after vaccination.

### Statistical analysis

The study was designed with a 90% power to detect the primary endpoint of non-inferiority in seroconversion of the fractional dose compared with the standard dose of the 17D-213 YF vaccine 28 days after vaccination. On the basis of previous data,^19^ we assumed 83% seroconversion and considered a one-sided test with level of significance of 2·5% and a non-inferiority margin of –0·17 (ie, –17 percentage point difference in seroconversion). The 17% non-inferiority margin is supported by a modelling study,^20^ in which fractional doses were shown to be beneficial in high-transmission areas if 80–90% efficacious and the fact that the HIV-positive population, constituting a minority within a larger population, have a smaller overall impact on herd immunity in the population if there is any reduction in immunogenicity.^20^, ^21^ The study allowed for 20% loss to follow-up or unevaluable participants. The overall sample size was 250. Analyses of all other efficacy and safety endpoints were considered as secondary outcomes and no adjustments were made for multiple comparisons.

There were three populations defined for analysis. The primary per-protocol population was defined as participants who gave a blood sample at baseline and at 28 (±3) days after vaccination, who were seronegative (PRNT_50_ <1:10) to yellow fever virus at baseline, for whom the eligibility criteria were appropriately applied and who adhered to the study protocol. Following the same criteria, a per-protocol population was also defined for the day 10 and day 365 visits. The intention-to-treat (ITT) population was defined as the group of participants who received a study vaccine and gave at least one post-vaccination blood sample. Immunogenicity endpoints were assessed for the subset of the ITT population who had pre-existing yellow fever neutralising antibodies (PRNT_50_ ≥1:10) at baseline. The safety population included all participants who were vaccinated with either the standard or fractional dose.

The number and percentage of participants who seroconverted, with their two-sided exact Clopper-Pearson 95% CIs, were summarised by dose. To assess non-inferiority for the primary outcome, we constructed a two-sided 95% CI around the point estimate of the difference between seroconversion rates in the fractional and standard dose groups using the Wilson score interval. Non-inferiority of the fractional dose was then concluded if the lower bound of the CI for the difference in seroconversion was greater than –17%.

Two-sided 95% CIs of the mean difference between the natural logarithm of GMT between the standard and fractional dose arms were produced using the *t*-distribution. These intervals were then transformed to show the ratio of the fractional dose to the standard dose for GMT. The same procedure was performed for GMFI. Reverse cumulative distributions were also produced.

The immunogenicity outcomes were assessed in the per-protocol and ITT populations. These analyses were repeated for 10 days and 365 days after vaccination (long term). Long-term assessment was done separately for individuals who were assessed within the planned study timelines (ie, 365 ±14 days) and those assessed outside of the study timelines (ie, >365 + 14 days).

Adverse events and serious adverse events were summarised as number and percentage by dose group. Safety outcomes were assessed in all vaccinated participants.

Analyses were performed using Stata version 15.0. Figures were produced in R version 3.6.2 and GraphPad Prism version 9.4.0.

This study is registered with ClinicalTrials.gov, NCT02991495.

### Role of the funding source

The funders of the study had no role in study design, data collection, data analysis, data interpretation, or writing of the report.

## Results

**Figure 1:**
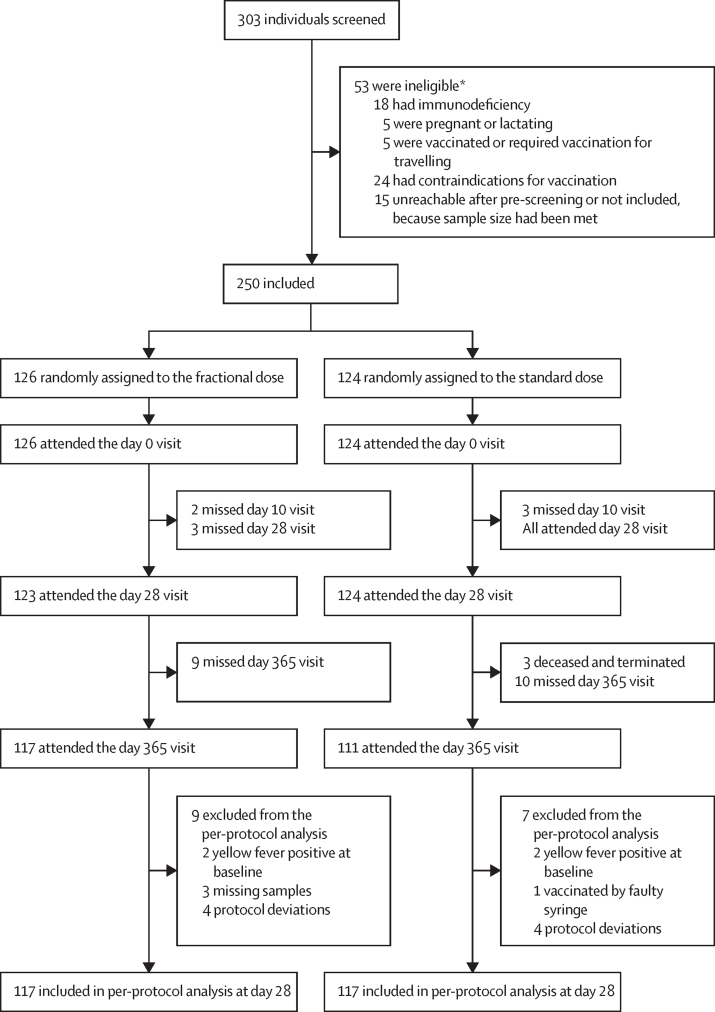
Study profile *Reasons are not mutually exclusive.

Between Jan 29 and May 17, 2019, 303 participants were screened, 53 were ineligible, and 250 were enrolled and randomly assigned to the standard dose (n=124) or the fractional dose (n=126; figure 1). The study had generally low attrition, with 5 participants missing their day 10 follow-up visit and three participants missing their day 28 follow up visit. Due to the COVID-19 pandemic and the restrictions put in place by the Kenyan government and Kenya Medical Research Institute, the study site was closed between April 6 and Aug 13, 2020, interrupting the scheduled 1-year follow-up visits. In accordance with ethical and regulatory bodies, study visits were re-scheduled and completed. As a result, 228 (91%) participants completed the last study follow-up visit; 83 (33%) were followed-up within their 1-year (±14 days) window, and 145 (58%) were followed up 3–5 months after their scheduled visit. 22 participants (9%) were lost to follow-up. The most frequent reason for attrition was safety uncertainties due to the COVID-19 pandemic. There were no discontinuations due to protocol violations.

The primary per-protocol analysis included 117 participants in the fractional dose group and 117 in the standard dose group (94% of participants). Exclusions from the per-protocol population were related to having detectable PRNT_50_ at baseline (n=4), protocol deviations related to missing CD4^+^ T-cell count at baseline (n=8), and vaccination with a faulty syringe (n=1). These participants were included in the ITT analysis. Additionally, there were 3 missed visits 28 days after vaccination.

The median age at enrolment was 36 years (IQR 28–45) and 129 (52%) were female (table 1). One participant reported a history of flavivirus infection (dengue). All participants were categorised as stage 1 HIV infection on the basis of the WHO clinical stage classification of HIV.^22^ The mean CD4^+^ T-cell count was 548·9 (SD 265·6) in the fractional dose group and 560·5 (SD 261·0) in the standard dose group. All participants were receiving antiretroviral therapy.

**Table 1:** Baseline characteristics

		**Fractional dose group (n=126)**	**Standard dose group (n=124)**
Median age at enrolment (IQR), years		36 (27–45)	38 (30–45)
Sex			
	Female	73 (58%)	56 (45%)
	Male	53 (42%)	68 (55%)
Seropositive to yellow fever at baseline*		2 (2%)	2 (2%)
Previous flavivirus infection		0	1 (<1%)
Geometric mean CD4^+^ T-cell count (SD), cells per mL		549 (266)	561 (261)

Data are n (%) unless otherwise specified. All participants were stage 1 HIV WHO clinical stage.

* Defined as 50% plaque reduction neutralisation test ≥10.

**Figure 2:**
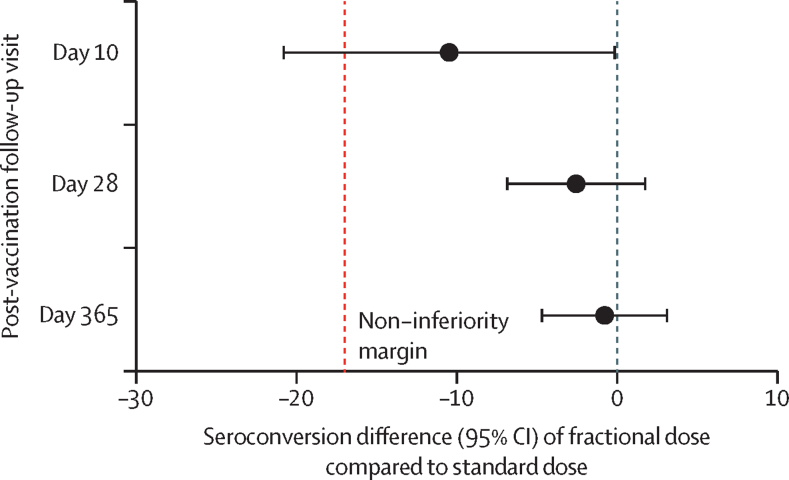
Non-inferiority of seroconversion of fractional doses compared with standard dose

28 days after vaccination, 112 (96%, 95% CI 90–99) of 117 participants in the fractional dose group and 115 (98%, 94–100) of 117 in the standard dose group seroconverted by PRNT_50_ (table 2). Numbers of patients who seroconverted were lower 10 days after vaccination (87 [74%, 65–81] of 118 in the fractional dose group and 96 [84%, 76–90] of 114 in the standard dose group) but were similar 365 days after vaccination (109 [97%, 92–100] of 112 and 104 [98%, 93–100] of 106). The difference in the seroconversion at 28 days (primary outcome) was –3% (95% CI –7 to 2); it was –10% (–21 to 0) at 10 days and –1% (–5 to 3) at 365 days (table 2; figure 2). Non-inferiority, defined as the lower bound of the CI greater than –17%, was met 28 days after vaccination and maintained 1 year after vaccination. The lower bound of the confidence interval for seroconversion at 10 days was –21%; however, this study was not powered to assess non-inferiority at day 10. The results at each timepoint were comparable using PRNT_90_ and in the per-protocol and the ITT populations (appendix pp 3,4).

**Table 2:** Immunological outcomes in the per-protocol population

		Seroconverted*, n/N (%, 95% CI)	**Seroconversion difference**†**, percentage points (95% CI)**	**Geometric mean titre (95% CI)**	**Geometric mean titre ratio**‡**(95% CI)**
Day 10		..	−10 (−21 to 0)	..	0·55 (0·35–0·86)
	Fractional dose	87/118 (74%, 65–81)	..	51 (37–69)	..
	Standard dose	96/114 (84%, 76–90)	..	92 (67–127)	..
Day 28		..	−3 (−7 to 2)	..	0·86 (0·53–1·41)
	Fractional dose	112/117 (96%, 90–99)	..	1391 (958–2019)	..
	Standard dose	115/117 (98%, 94–100)	..	1613 (1163–2236)	..
Day 365		..	−1 (−5 to 3)	..	0·71 (0·45–1·13)
	Fractional dose	109/112 (97%, 92–100)	..	846 (599–1194)	..
	Standard dose	104/106 (98%, 93–100)	..	1191 (869–1634)	..

* Seroconversion is defined as ≥4-fold increase in neutralising antibody titre at each timepoint from baseline.

† Seroconversion Difference=Fractional–Standard.

‡ Geometric mean titre ratio=Fractional ÷ Standard.

At 28 days, the GMT was 1391 (958–2019) in the fractional dose group and 1613 (1163–2236) in the standard dose group and GMFI was 278 (192–404) and 323 (233–447; table 2; appendix p 4). GMTs and GMFIs were lower 10 days after vaccination compared with 28 days, and titres were significantly lower in the fractional dose group compared with standard dose group (51 [37–69] *vs* 92 [67–127]; p=0·0093). The high GMTs and GMFIs seen at 28 days persisted at long-term follow-up for the participants that were followed up as per protocol at day 365 (±14) and those followed up beyond the window period (appendix p 4). There was some decline in GMTs and GMFIs between 28 days and the long-term follow-up. The GMTs and GMFIs were consistently lower by PRNT_90_ compared with PRTN_50_, but differences between doses were comparable in the per-protocol analysis and the ITT population across all timepoints (appendix pp 4–7). Very few participants were seropositive for yellow fever at baseline (table 1), and the ratio of GMFI comparing the fractional doses with standard doses was similar to the comparison using GMTs (appendix p 4). Titres in the fractional dose group were consistently lower than in the standard dose group, but only the difference at 10 days was significant by log-rank test (p=0·016; appendix p 9).

110 participants reported at least one adverse event occurring within the 28 days following vaccination. 49 (39%) of 126 participants in the fractional dose group and 61 (49%) of 124 in the standard dose group had an adverse event, accounting for a total of 187 adverse events. There were no adverse events immediately after vaccination. 18 (14%) of 126 participants in the fractional dose group and 33 (27%) of 124 in the standard dose group were classified as having an adverse event related to the study vaccine (table 3). The most common adverse events were headache (n=31 [12%]), fatigue (n=23 [9%]), myalgia (n=23 [9%]), and cough (n=14 [6%]; appendix pp 9–10). The reported adverse events were either mild (182 [97%] of 187 adverse events) or moderate (5 [3%] of 187) and were self-limiting (table 3). There were no major differences in adverse events by sex, age or CD4^+^ T-cell count. There were seven serious adverse events reported throughout the study, none of which were classified as related to vaccination (table 3). These serious adverse events included acute kidney injury, blunt trauma, subdural hematoma, tibia fracture, infection of a burn wound, and sudden death and were reported to the ethical and regulatory bodies.

**Table 3:** Adverse events up to 28 days after vaccination and serious adverse events throughout follow-up

		**Fractional dose group (n=126)**	**Standard dose group (n=124)**
**Overall**			
At least one adverse event		49 (39%)	61 (49%)
Vaccine-related adverse events		18 (14%)	33 (27%)
Severity			
	Mild	48 (38%)	59 (48%)
	Moderate	1 (<1%)	2 (2%)
	Severe	0	0
	Life threatening	0	0
Serious adverse events		2 (2%)	5 (4%)
**By MedDRA system organ classes and preferred terms**			
General disorders and administration site conditions		0	2 (2%)
	Death	0	1 (<1%)
	Sudden death, cause unknows	0	1 (<1%)
Infections and infestations		1 (<1%)	0 (0%)
	Burn infection	1 (<1%)	0 (0%)
Injury, poisoning, and procedural complaints		1 (<1%)	2 (2%)
	Subdural haematoma	0	1 (<1%)
	Tibia fracture	0	1 (<1%)
	Trauma	1 (<1%)	0
Renal and urinary disorders		0 (0%)	1 (<1%)
	Acute kidney injury secondary to tenofovir nephropathy	0 (0%)	1 (<1%)

Data are n (%).

## Discussion

Mass pre-emptive and reactive vaccination campaigns in yellow fever endemic regions have substantial benefits in tackling epidemics.^23^ In recent outbreaks in parts of Africa and South America, fractional doses (one-fifth of the standard dose) were used as a dose sparing strategy. In these settings, about 1–10% of the population is infected with HIV.^4^ Assessment of the efficacy of fractional doses of yellow fever vaccines in HIV-infected individuals is therefore necessary.

To our knowledge, this substudy is the first randomised controlled non-inferiority trial assessing fractional doses and standard doses of a yellow fever vaccine in a population of HIV-infected adults in Africa.^24^ This study affirms that fractional doses can be used in HIV-infected adults with no symptoms of current clinical immunosuppression and CD4^+^ T-cell count of at least 200 cells per mL. One-fifth of the standard dose of the 17D-213 YF vaccine manufactured by the Chumakov Institute of Poliomyelitis and Viral Encephalitidis in Russia, was safe and immunogenically non-inferior to the standard dose in HIV-infected individuals at 28 (±3) days after vaccination. These results are consistent with the evidence from the preceding study assessing immunogenicity and safety in the general adult population in Kenya and Uganda^3^ and the results from an observational study implemented in the Democratic Republic of the Congo during a fractional dose campaign implemented as part of the response to an outbreak.^25^

There was good compliance with the protocol among trial participants, with an attrition rate of 9% at long-term follow-up. The baseline positivity for yellow fever as measured by PRNT_50_ was 2%. Yellow fever virus transmission has not been reported on the coast of Kenya, and the positivity could be due to participants who had been previously vaccinated or cross-reactivity with other flaviviruses circulating in the coast of Kenya, such as dengue virus.^6^

A single dose of the fractional dose (one-fifth of the standard dose) induced robust immunity with high neutralising antibody titres similar to the standard dose. The primary outcome, non-inferiority of seroconversion at 28 days after vaccination as measured by PRNT_50_, was demonstrated and maintained at long-term follow-up. GMTs were comparable for both study groups with overlapping CIs, although the standard dose induced slightly higher GMTs across all study-specific timepoints compared with fractional doses. Seroconversion rates and GMTs were lower 10 days after vaccination compared with the other timepoints. According to the WHO International Health Regulations guidance, vaccinees are considered protected at 10 days after vaccination. In this study, 74% of participants in the fractional dose group seroconverted at 10 days compared with 84% in the standard dose group, with large overlapping CIs. These seroconversion rates were higher than what we have previously found in the general adult population, with seroconversion rates of 53% with fractional doses and 61% with standard doses of the 17D-213 vaccine^3^ and closer to the expected 80–90% seroconversion previously reported.^26^ Nevertheless, better understanding of the practical implications of a potential delay in the immunological response in the response to an outbreak is needed. Detailed time course studies and modelling are warranted. Although there are concerns for low seroconversion rates at day 10 in the context of pre-emptive and reactive vaccination campaigns during an outbreak, fractional doses have previously been used successfully as part of the response to control outbreaks.^25^ Nevertheless, the practical significance of the lower seroconversion at day 10 might need more detailed time course studies and modelling work to determine any potential impact on effectiveness of fractional dosing during vaccination campaigns in response to outbreaks.

There were local and systemic adverse events reported in both vaccine dose groups, with participants in the fractional doses group reporting fewer events. There were no safety concerns, because all adverse events were either mild or moderate in severity and resolved without sequelae, and none of the serious adverse events were related to vaccine administration or participation in the study. The safety profile was consistent with previous studies in HIV-infected individuals.^27^ There have been safety concerns in individuals with very low CD4^+^ T-cell counts (<100 cells per mL) owing to reports of fatal cases of yellow fever encephalitis after vaccination and increased risks of developing yellow fever vaccine-associated viscerotropic diseases and neurotropic disease has been documented in this sub-group.^28^ In our study, all participants had CD4^+^ T-cell counts of more than 200 cells per mL.

Consistent with the results of the preceding study,^3^ there was evidence of some decline in GMT between 28 days and 365 days after vaccination in this study, although seroconversion rates were high at both timepoints. Antibody titres were much lower in the HIV-infected population, with 3–4 times lower titres in both the fractional and standard dose arms, when compared with the titres observed in the general adult population in our preceding study.^3^ Although more studies are needed to better understand the immune response in people living with HIV, the lower antibody titres in this population relative to HIV-negative individuals might be an early indication of the need for a booster vaccine for long-term immunity. A systematic review and meta-analysis^29^ assessing duration of protection after standard yellow fever vaccination has shown lower antibody titres and a more rapid decrease 10 years after vaccination, in people with HIV infections compared with healthy controls. Studies looking at the long-term protection of fractional doses are needed to better frame the use of fractional doses. Further studies could also include assessments of T cell immunity, and its association with vaccine dose, vaccine viraemia, and HIV viral load, among others. A systems immunology approach stratified by dosing group in different populations (eg, HIV-negative individuals, HIV-infected individuals, and children) might provide new insights into the immune response to fractional doses of yellow fever vaccines.

Individuals with CD4^+^ T-cell counts of more than 200 cells per mL have been shown to have adequate immunological responses comparable to HIV-negative individuals.^8^, ^9^ Although there is high global coverage of antiretroviral therapy in HIV-infected individuals,^4^ some resource-limited settings still have delayed and suboptimal testing for HIV, and HIV-infected individuals living there are often late in receiving antiretroviral therapy.

This study has several limitations. First, in epidemic settings, HIV-infected people might have varying disease severity and might be severely immunocompromised. However, we did not assess individuals with CD4^+^ T-cell counts of less than 200 cells per mL. Our results are therefore not directly generalisable to individuals with uncontrolled HIV infection. Second, this study was limited to about 12–17 months of follow-up for longevity, so durability of the immune response beyond 17 months will need further study. Third, we only describe humoral immunity in terms of functional antibodies. Our study therefore does not describe cellular immunity, which might also determine the immunological protection of vaccination.^30^ Finally, the definition of fractional (one-fifth) is difficult to generalise, because vaccine manufacturers release yellow fever vaccines at varying potencies, which often exceed the minimum standard dose recommendation by far more than five times. Therefore, determination of the lower doses of vaccines that are immunogenic is needed.

In conclusion, fractional doses of the 17D-213 yellow fever vaccine are safe, effective, and immunologically non-inferior to standard doses in HIV-infected individuals on antiretroviral therapy with CD4^+^ T-cell counts of at least 200 cells per mL. The evidence presented here is consistent with evidence in the general adult population, in which fractional doses of the four WHO-prequalified vaccines were shown to not be worse than the standard doses. The results of this study provide information on the applicability of fractional doses in populations with a high HIV prevalence.

## Data sharing

Data collected for the study, including deidentified participant data, data dictionary, and additional related documents, such as study protocol and statistical analysis plan, will be made available to others upon request to dgc@kemri-wellcome.org, following the Kenya Medical Research Institute's data sharing policy and in accordance with WHO statement on public disclosure of clinical trial results.

## Declaration of interests

We declare no competing interests.
