# Supplementary material for: Immunogenicity and safety of fractional doses of 17D-213 yellow fever vaccine in HIV-infected people in Kenya (YEFE): a randomised, double-blind, non-inferiority substudy of a phase 4 trial
Source: Lancet Infect Dis. 2023 Aug;23(8):974–82. doi: 10.1016/S1473-3099(23)00114-7 (PMC10371873; doi:10.1016/S1473-3099(23)00114-7)
Supplement: Supplementary appendix [file mmc1.pdf]

THE LANCET
Infectious Diseases

Supplementary appendix

This appendix formed part of the original submission and has been peer reviewed. We post it as supplied by the authors.

Supplement to: Kimathi D, Juan-Giner A, Orindi B, et al. Immunogenicity and safety of fractional doses of 17D-213 yellow fever vaccine in HIV-infected people in Kenya (YEFE): a randomised, double-blind, non-inferiority substudy of a phase 4 trial. *Lancet Infect Dis* 2023; published online April 28. https://doi.org/10.1016/S1473-3099(23)00114-7.

Immunogenicity and safety of fractional doses of 17D-213 yellow fever vaccine among HIV-infected persons in Kenya: a randomised, double-blind, non-inferiority trial.

Derick Kimathi, PhD[1,2]*, Aitana Juan-Giner, MSc[3], Benedict Orindi, PhD[1], Kyra H. Grantz, BA[4,5,6], Ndeye S Bob, PhD[7], Stanley Cheruiyot, BSc[1], Mainga Hamaluba, MD[1,2], Naomi Kamau, BSc[1], Gamou Fall, PhD[7], Moussa Dia, MSc[7], Moses Mosobo, BSc[1], Felix Moki, Dip[1], Kenneth Kiogora, Dip[1], Oscar Chirro, MSc[1], Alexander Thiong'o, BSc[1], Jane Mwendwa, BSc[1], Andrew Guantai, Dip [1], Henry K. Karanja, MSc[1], John Gitonga, BSc[1], Daisy Mugo, BSc[1], Kelly Ramko, MSc [1], Ousmane Faye, PhD[7], Prof Eduard J. Sanders, PhD[1,2], Rebecca F Grais, PhD[3], Prof Philip Bejon, PhD FMedSc [1,2], Prof George M. Warimwe, PhD[1,2#].

[1] Kenya Medical Research Institute (KEMRI) – Wellcome Trust Research Programme, Kilifi, Kenya

[2] Centre for Tropical Medicine & Global Health, University of Oxford, UK

[3] Epicentre, Paris, France

[4] Department of Biology, University of Florida

[5] Emerging Pathogens Institute, University of Florida

[6] Department of Epidemiology, Johns Hopkins Bloomberg School of Public Health

[7] Institut Pasteur Dakar, Senegal

#Corresponding author: George Warimwe; KEMRI-Wellcome Trust Research Programme CGMRC, PO Box 230-80108, Kilifi, Kenya; Email gwarimwe@kemri-wellcome.org

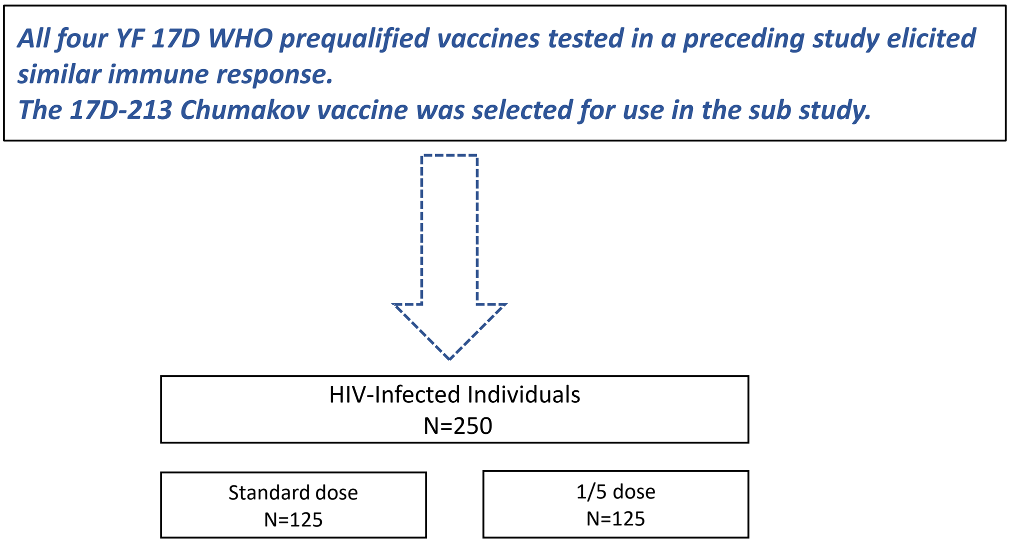

Figure S1 of the study design.

The vaccine

The vaccine potency was confirmed at the National Institute for Biological Standards and Control (NIBSC), a biological standardisation agency in the UK and is presented in table S1. The potency tests were done with and without pre-incubation at 37ºC. These tests are recommended by the WHO to establish the stability of vaccines and the status of the vaccine infectivity when exposed to high temperatures.

Table S1 Potency of the YF vaccine lot 598.

**Product**	**Test**	**Assay 1**	**Assay 2**	**Assay 3**	**Mean (SD) (log IU/dose)**	**(IU/dose)**
Yellow Fever Vaccine lot 598 FSUE, Russia	Potency test	4.88	4.85	4.77	**4.83** (0.06)	**~67,608 IU/dose**

The standard dose of the vaccine was shown to have a potency of 4.83 logIU/dose (~67,608IU/dose). The fractional dose (a fifth) of the standard dose corresponds to ~13,521IU/dose.

Table S2 Summary of Screening and Enrolment

Description	No. of individuals
Participants screened	303
Participants deemed ineligible*	53
Immunodeficiency	18
Pregnant/ Lactating	5
Allergy to egg proteins	5
History of YF vaccination	4
Unable to complete follow-up	0
Acute febrile illness	0
Requiring vaccination for travel purposes	1
Refusal to participate	0
Other criteria**	22
Participants vaccinated	250

**Participants could fail to meet more than one eligibility criterion.*
***lost to follow-up after pre-screening (n=14), on hepatotoxic drugs (n=5), anaemia with TB symptoms (n=1), sample size completed (n=1), mental unwellness (n=1).*

Immunogenicity Analysis

Table S3. Immunological outcomes using $PRNT_{50}$ in the per-protocol population*

	Seroconverted, n/N (%, 95% CI)	Seroconversion difference	GMT (95% CI)	GMT ratio	GMFI titre (95% CI)	GMFI ratio
Day 10						
Fractional dose	87/118 (73.7%, 64.8-81.4)		50.6 (37–69.2)		10 (7–14)	
Standard dose	96/114 (84.2%, 76.2-90.4)	-10.48 (-20.87– -0.1)	92 (66.5–127.4)	0.55 (0.35–0.86)	18 (13–25)	0.55 (0.35–0.86)
Day 28						
Fractional dose	112/117 (95.7%, 90.3-98.6)		1391 (958–2019)		278 (192–404)	
Standard dose	115/117 (98.3%, 94.0-99.8)	-2.56 (-6.92–1.79)	1613 (1163–2236)	0.86 (0.53–1.41)	323 (233–447)	0.86 (0.53–1.41)
Day 365 (all)						
Fractional dose	109/112 (97.3%, 92.4-99.4)		846 (599–1194)		169 (120–239)	
Standard dose	104/106 (98.1%, 93.4-99.8)	-0.79 (-4.75–3.16)	1191 (869–1634)	0.71 (0.45–1.13)	238 (174–327)	0.71 (0.45–1.13)
Day 365 (-/+14)						
Fractional dose	37/38 (97.4%, 86.2-99.9)		956 (534–1711)		191 (107–342)	
Standard dose	37/37 (100%, 90.5-100)	-2.63 (-7.72–2.46)	2658 (1664–4246)	0.36 (0.17–0.75)	532 (333–849)	0.36 (0.17–0.75)
Day >379						
Fractional dose	72/74 (97.3%, 90.6-99.7)		794 (513–1230)		159 (103–246)	
Standard dose	67/69 (97.1%, 89.9-99.6)	0.20 (-5.22–5.61)	775 (527–1138)	1.02 (0.58–1.83)	155 (105–228)	1.02 (0.58–1.83)

*Seroconversion difference= Fractional – standard; GMT = Geometric mean titre; GMFI = Geometric mean titre fold increase; ratio=Fractional/standard

Table S4: Immunological outcomes using $PRNT_{90}$ in the per-protocol population

Dose Level	Per-protocol population			
	**% SC (95% CI)**	**GMT (95% CI)**	**GMFI (95% CI)**	**Ratio: GMT (Fractional/ Standard) (95% CI)**
**Immunological parameters using $PRNT_{90}$**				
**Day 10 ($PRNT_{90}$)**				
Standard dose (n=114)	30 (22, 39)	10.1 (8.6, 11.9)	2.0 (1.7, 2.4)	0.78 (0.64, 0.95)
Fractional dose (n=118)	18 (11, 26)	7.9 (7.0, 8.9)	1.6 (1.4, 1.8)	
**Day 28 ($PRNT_{90}$)***				
Standard dose (n=117)	82 (74, 88)	69.0 (50.8, 93.8)	13.8 (10.2, 18.8)	0.98 (0.64, 1.5)
Fractional dose (n=117)	80 (71, 86)	67.4 (49.8, 91.1)	13.5 (10, 18.2)	
**Day 365 ($PRNT_{90}$)**				
Standard dose (n=106)	84 (76, 90)	46.5 (37.5, 57.7)	9.3 (7.5, 11.5)	0.87 (0.63, 1.21)
Fractional dose (n=112)	79 (70, 86)	40.5 (31.5, 52)	8.1 (6.3, 10.4)	

* The differences in the seroconversion rates for the fractional dose compared to the standard dose using $PRNT_{90}$ titres were -3% (95% CI -13 to 8%) at day 28

Table S5: Immunological outcomes in the ITT population by $PRNT_{50}$ and $PRNT_{90}$

Dose Level	ITT population analysis			
	**% SC (95% CI)**	**GMT (95% CI)**	**GMFI (95% CI)**	**Ratio: GMT (Fractional/ Standard) (95% CI)**
**Immunological parameters using $PRNT_{50}$**				
**Day 10 ($PRNT_{50}$)**				
Standard dose (n=121)	85 (78, 91)	98.3 (71.6, 135)	18.3 (13.3, 25)	0.52 (0.34, 0.8)
Fractional dose (n=124)	75 (66, 82)	51.2 (37.9, 69)	9.8 (7.3, 13.3)	
**Day 28 ($PRNT_{50}$)**				
Standard dose (n=124)	98 (94, 100)	1610 (1172, 2211)	299 (212, 422)	0.91 (0.57, 1.47)
Fractional dose (n=123)	96 (91, 99)	1465 (1024, 2096)	282 (196, 404)	
**Day 365* ($PRNT_{50}$)**				
Standard dose (n=111)	98 (94, 100)	1225 (910, 1648)	219 (157, 306)	0.83 (0.53, 1.28)

Fractional dose (n=117)	97 (93, 100)	1012 (733, 1397)	182 (129, 255)	
**Immunological parameters using $PRNT_{90}$**				
**Day 10 ($PRNT_{90}$)**				
Standard dose (n=121)	30 (22, 39)	10.4 (8.8, 12.3)	2 (1.8, 2.4)	0.76 (0.62, 0.92)
Fractional dose (n=124)	17 (11, 25)	7.9 (7, 8.8)	1.6 (1.4, 1.8)	
**Day 28 ($PRNT_{90}$)**				
Standard dose (n=124)	82 (74, 86)	70 (52, 94)	13.8 (10.2, 18.5)	0.98 (0.65,1.48)
Fractional dose (n=123)	81 (72, 87)	68.7 (51.5, 91.7)	13.7 (10.3, 18.3)	
**Day 365* ($PRNT_{90}$)**				
Standard dose (n=111)	83 (75, 89)	46.5 (37.6, 57.4)	9.1 (7.4, 11.3)	0.96 (0.69, 1.33)
Fractional dose (n=117)	80 (71, 86)	44.5 (34.5, 57.4)	8.9 (6.9, 11.5)	

Table S6 Non-inferiority of seroconversion rate in fractional vs. full dose of YF vaccine with follow-up at 365 (-/+ 14) days in HIV+ in PP and ITT populations, by $PRNT_{50}$ and $PRNT_{90}$

**$PRNT_{50}$**					
	**Fractional dose**		**Standard dose**		**Difference (Fractional - Standard)**
	No. SC	% SC (95% CI)	No. SC	% SC (95% CI)	(95% CI)
PP population	37/38	97 (86, 100)	37/37	100 (90, 100)	-3 (-8, 2)
ITT population	42/43	98 (88, 100)	40/40	100 (91, 100)	-2 (-7, 2)
**$PRNT_{90}$**					
	**Fractional dose**		**Standard dose**		**Difference (Fractional - Standard)**
	No. SC	% SC (95% CI)	No. SC	% SC (95% CI)	No. SC
PP population	31/38	82 (66, 92)	31/37	84 (68, 94)	-2 (-19, 15)
ITT population	36/43	84 (69, 93)	33/40	83 (67, 93)	1 (-15, 17)

Table S7 Non-inferiority of seroconversion rate in fractional vs. full dose of YF vaccine with follow-up at >365 + 14 days in HIV+ in PP and ITT populations, by $PRNT_{50}$ and $PRNT_{90}$

**$PRNT_{50}$**					
	**Fractional dose**		**Standard dose**		**Difference (Fractional - Standard)**
	No. SC	% SC (95% CI)	No. SC	% SC (95% CI)	(95% CI)
PP population	72/74	97 (91, 100)	67/69	97 (90, 100)	0.20 (-5.22, 5.61)
ITT population	72/74	97 (91, 100)	69/71	97 (90, 100)	0.10 (-5.20, 5.40)
**$PRNT_{90}$**					
	**Fractional dose**		**Standard dose**		**Difference (Fractional - Standard)**
	No. SC	% SC (95% CI)	No. SC	% SC (95% CI)	No. SC
PP population	57/74	77 (66, 86)	58/69	84 (73, 92)	-7 (-20, 6)
ITT population	57/74	77 (66, 86)	59/71	83 (72, 91)	-6 (-19, 7)

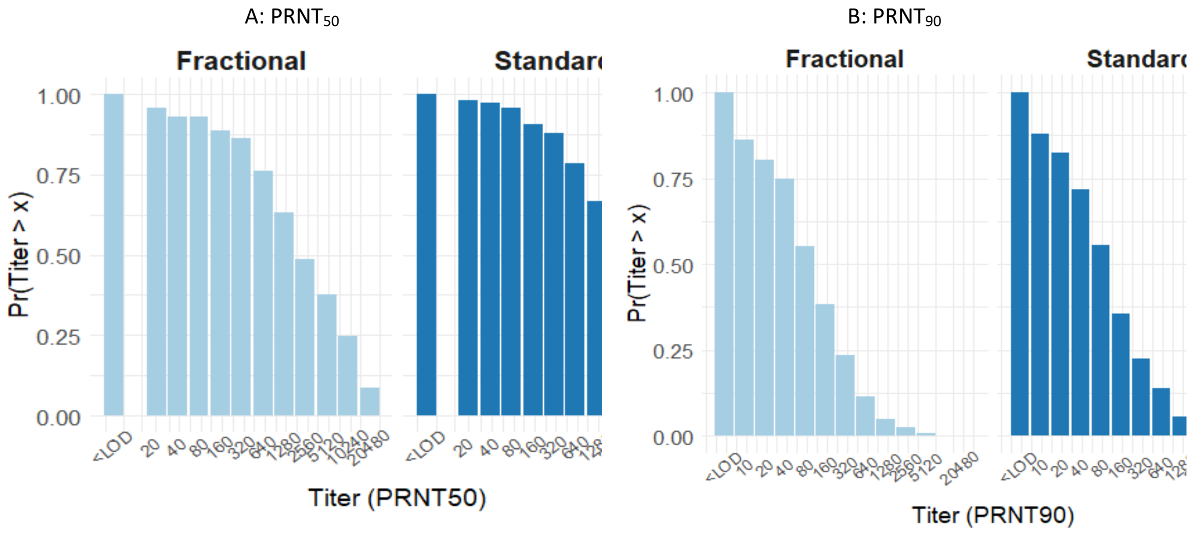

Figure S2 Reverse cumulative distributions of GMT at Day 28 for HIV+ PP, by vaccine dose and PRNT$_{50}$ (A) and PRNT$_{90}$ (B)

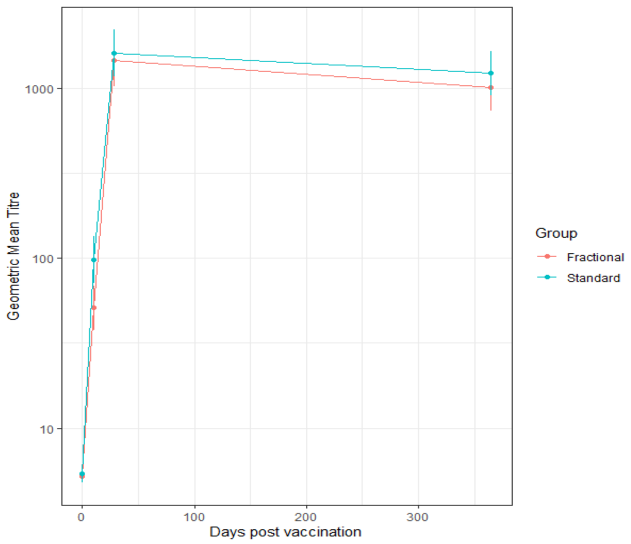

Figure S3 GMT at Day 0, 10, 28, and 365 for HIV+ ITT population, by vaccine dose and by PRNT$_{50}$

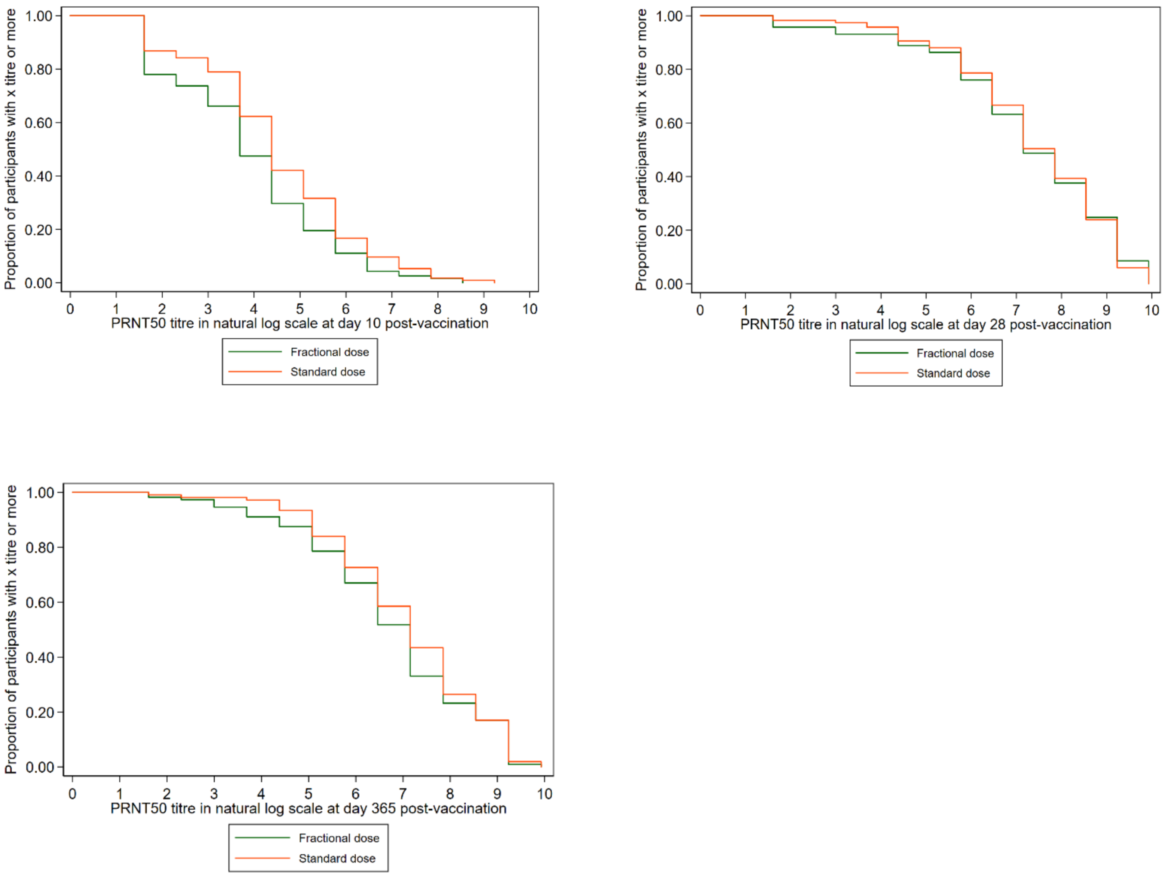

Figure S4 Reverse cumulative distribution plots of titres at day 10, day 28 and day 365 post-vaccination by PRNT$_{50}$ for the PP population. Titres were significantly different at day 10 (p=0.016), but not at day 28 (p=0.964) nor day 365 (p=0.338).

Safety analysis

Table S7 Adverse events up to Day 28 post-vaccination by MedDRA coding (SOC and PT) by study arm.

**System Organ Class and Preferred Term**	**Fractional dose**	**Standard dose**
n (%) with ≥ 1 AE	N=126	N=124
**Eye Disorders**	**1(0.8)**	**0(0)**
Eye disorder	1 (0.8)	0 (0)
**Gastrointestinal disorders**	**10 (8)**	**14 (11.2)**
Abdominal discomfort	0 (0)	2 (1.6)
Abdominal pain	4 (3.2)	9 (7.3)
Anorectal disorder	1 (0.8)	0 (0)
Nausea	3 (2.4)	1 (0.8)

Stomatitis	1 (0.8)	0 (0)
Diarrhoea	1 (0.8)	2 (1.6)
**General disorders and administration site conditions**	**16 (12.7)**	**17 (13.7)**
Fatigue	12 (9.5)	11 (8.9)
Vaccination site discomfort	0 (0)	2 (1.6)
Pyrexia	4 (3.2)	4 (3.2)
**Infections and infestations**	**8 (6.3)**	**11 (8.9)**
Dysentery	0 (0)	1 (0.8)
Fungal skin infection	1 (0.8)	1 (0.8)
Furuncle	1 (0.8)	0 (0)
Gastroenteritis	1 (0.8)	3 (2.4)
Lower respiratory tract infection	1 (0.8)	1 (0.8)
Lower urinary tract symptoms	0 (0)	1 (0.8)
Staphylococcal skin infection	1 (0.8)	0 (0)
Tonsillitis	2 (1.6)	1 (0.8)
Urinary tract infection	1 (0.8)	1 (0.8)
Vaginal candidiasis	0 (0)	1 (0.8)
Wound sepsis	0 (0)	1 (0.8)
**Ear and labyrinth disorders**	**1 (0.8)**	**1 (0.8)**
External ear disorder	1 (0.8)	0 (0)
Ear pain	0 (0)	1 (0.8)
**Injury, poisoning and procedural complications**	**0 (0)**	**2 (1.6)**
Injury	0 (0)	2 (1.6)
**Musculoskeletal and connective tissue disorders**	**18 (14.3)**	**18 (14.5)**
Arthralgia	8 (6.3)	5 (0.4)
Myalgia	10 (7.9)	13 (10.5)
**Nervous system disorders**	**16 (12.7)**	**24 (19.4)**
Dizziness	2 (1.6)	7 (5.6)
Headache	14 (11.1)	17 (13.7)
**Reproductive system and breast disorders**	**0 (0)**	**1 (0.8)**
Dysfunctional uterine bleeding	0 (0)	1 (0.8)
**Respiratory, thoracic and mediastinal disorders**	**10 (7.9)**	**15 (12.1)**
Asthma	1 (0.8)	0 (0)
Cough	5 (0.4)	9 (7.3)
Rhinorrhoea	4 (3.2)	6 (4.8)
**Skin and subcutaneous tissue disorders**	**2 (1.6)**	**2 (1.6)**
Dermatitis	1 (0.8)	1 (0.8)
Skin irritation	0 (0)	1 (0.8)
Skin lesion	1 (0.8)	0 (0)

Data and Safety Monitoring Board (DSMB) composition and meetings

The DSMB was composed of 3 members independent to the study who collectively had experience in the management of patients in Africa, vaccinology, immunology, and global policy as well as the conduct and monitoring of randomised clinical trials. Members were the same for the entire trial (this comprises the evaluation of the safety and immunological non-inferiority of the 4 WHO-prequalified YF vaccines in adults and the sub-studies evaluating one of the YF vaccines in children and HIV-infected adults).

The DSMB was responsible for safeguarding the interests of trial participants and assessing the immunogenicity and safety of the interventions during the trial. The responsibilities were exercised by providing recommendations about stopping, continuing, or modifying the trial.

The DSMB received regular information about the trial implementation.

The DSMB reviewed immunogenicity and safety data during a meeting held on 26th September 2018 and provided the recommendations to continue with the sub-studies.
